# Refractory Bilateral Tubo-Ovarian Abscesses in a Patient with Iatrogenic Hypogammaglobulinemia

**DOI:** 10.3390/diagnostics13223478

**Published:** 2023-11-19

**Authors:** Elizabeth J. Klein, Nouf K. Almaghlouth, Gabriela Weigel, Dimitrios Farmakiotis, Erica Hardy

**Affiliations:** 1 Division of Infectious Diseases, The Warren Alpert Medical School of Brown University, Providence, RI 02903, USA; elizabeth_klein@brown.edu (E.J.K.); nalmaghlouth@lifespan.org (N.K.A.); dimitrios.farmakiotis@lifespan.org (D.F.); 2 Department of Obstetrics and Gynecology, The Warren Alpert Medical School of Brown University, Providence, RI 02903, USA; gweigel@wihri.org; 3 Divisions of Obstetric Medicine and Infectious Diseases, Women and Infants Hospital, Providence, RI 02905, USA

**Keywords:** *Ureaplasma*, Mollicutes, hypogammaglobulinemia, cell-free DNA, next-generation sequencing

## Abstract

Genital mycoplasmas are sexually transmitted Mollicutes with a high prevalence of urogenital tract colonization among females of reproductive age. Current guidelines recommend against routine screening for these organisms, since their role in the pathogenesis of pelvic inflammatory disease and tubo-ovarian abscesses (TOAs) remains unclear. However, genital mycoplasmas harbor pathogenic potential in immunocompromised hosts, especially patients with hypogammaglobulinemia. It is important to identify such infections early, given their potential for invasive spread and the availability of easily accessible treatments. We present a young adult female with multiple sclerosis and iatrogenic hypogammaglobulinemia, with refractory, bilateral pelvic inflammatory disease and TOAs due to *Ureaplasma urealyticum*, identified as a single pathogen via three distinct molecular tests. To our knowledge, this is the second case of TOAs caused by *U. urealyticum* in the literature, and the first diagnosed by pathogen cell-free DNA metagenomic next-generation sequencing in plasma.

## 1. Introduction

Genital mycoplasmas (*Ureaplasma* and *Mycoplasma* spp.) are bacteria of the urogenital tract flora and can be acquired through sexual contact [1]. They are characterized by their comparatively small genomes and lack of a cell wall, yielding them unable to be visualized on Gram stain nor grow on conventional culture media [2]. The prevalence of *U. urealyticum* in nonpregnant women of reproductive age is estimated at 7.6–28.4% [3]. This wide range is explained in part by discrepancies in sample source (e.g., urine, cervix, peritoneum) and method of identification (e.g., PCR, culture, antigen).

The contribution of *Ureaplasma* infections to lower genital tract pathology, including pelvic inflammatory disease, cervicitis, and genital discomfort, is inconclusive [3,4,5]. Although PCR testing for these species is widely available, routine screening is not recommended, since asymptomatic carriage of *Ureaplasma* spp. is common [6,7]. Nonetheless, it is important to consider how the risk–benefit tradeoff for testing and treatment shifts in the setting of immunosuppression. There have been multiple case studies of *U. urealyticum* infections in immunocompromised children and adults in the setting of hematologic malignancy [8], autoimmune disease [9,10], and organ transplant [11,12]. The majority of these cases are reported in patients with congenital or iatrogenic hypogammaglobulinemia [13]. 

Herein, we present a case of refractory, bilateral tubo-ovarian abscesses (TOAs) caused by *U. urealyticum* in a nonpregnant hypogammaglobulinemic female of reproductive age. To our knowledge, only one similar case has been reported [9], and this organism is not considered an established cause of TOA. 

## 2. Clinical Case Description

**Figure 1:**
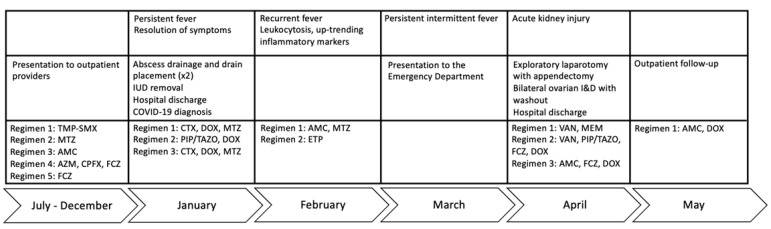
Clinical course. Antibiotic treatment was dictated by clinician judgement. Standard pelvic inflammatory disease regimens were started to target the most common causes, before coverage was broadened to include resistant enteric organisms. TMP-SMX, trimethoprim/sulfamethoxazole; AZM, azithromycin; CPFX, ciprofloxacin; FCZ, fluconazole; CTX, ceftriaxone; DOX, doxycycline; MTZ, metronidazole; PIP/TAZO, piperacillin/tazobactam; AMC, amoxicillin/clavulanate; ETP, ertapenem; VAN, vancomycin.

**Figure 2:**
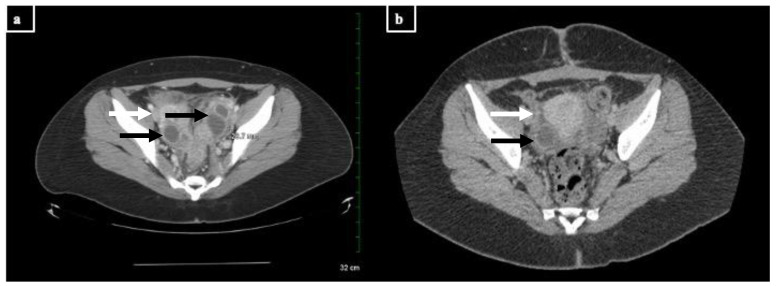
CT images of the TOAs before and after treatment with doxycycline and drainage (scale: 32 cm, measured dimension 28.7 mm at this level). Cystic structures are denoted with black arrows; inflammatory stranding is denoted with white arrows. (**a**) Representative image from the patient’s CT scan upon admission in March. There are multi-cystic structures with rim enhancement and inflammatory stranding in the bilateral ovaries consistent with bilateral residual or recurrent TOAs. (**b**) Representative image from the patient’s CT scan after treatment in June. There is a 2.0 cm benign cyst in the right ovary as well as moderate inflammatory stranding in the pelvis, consistent with recent pelvic inflammatory disease.

A 20-year-old female with multiple sclerosis (MS) receiving rituximab twice annually for six years presented to an outside hospital with fever and left-sided abdominal pain. She had been sexually active with one partner, with reported use of condoms and an intrauterine device (IUD) for contraception. She received courses of oral antibiotics as an outpatient prior to her initial presentation, including trimethoprim/sulfamethoxazole, metronidazole, amoxicillin/clavulanate, azithromycin, ciprofloxacin, and fluconazole (Figure 1). Radiographic imaging demonstrated a left, multi-cystic adnexal mass measuring 9.5 × 5.4 × 4.7 cm consistent with a large left TOA (Figure 2). The patient underwent computed tomography (CT)-guided aspiration of the collection with purulent fluid drainage and no growth on conventional bacterial cultures. She received intravenous ceftriaxone, doxycycline, and metronidazole; her IUD was removed. She continued to have persistent fevers, prompting the placement of a temporary drain and escalation from ceftriaxone and metronidazole to piperacillin/tazobactam. She was discharged with improved symptoms on ceftriaxone 2 g intravenous daily for three weeks, doxycycline 100 mg twice daily, and metronidazole 500 mg twice daily. The latter two were discontinued after ten days of treatment. Testing for human immunodeficiency virus (HIV), syphilis, and gonorrhea/chlamydia were negative. She was diagnosed with COVID-19 and was unable to complete the planned course of intravenous ceftriaxone at an infusion clinic; therefore, she received oral amoxicillin/clavulanate 875–125 mg twice daily for 14 days and metronidazole. However, she had recurrent fevers and her antimicrobial therapy was changed to ertapenem, with questionable improvement in her symptoms and intermittent episodes of fever. 

After completion of several weeks of ertapenem, she presented to our hospital with fever; repeat CT scan demonstrated persistent, enlarging adnexal collections. She underwent two diagnostic laparoscopies, two washout procedures, exploratory laparotomy with appendectomy, and bilateral ovarian incision and drainage with a placement of bilateral drains, complicated by wound dehiscence with purulent discharge that was sent to the University of Washington for multiplex PCR. An appendectomy was performed, given intra-operative findings concerning for a nidus of infection at the appendix. On a manual exploration of the pelvis, the appendix was found to be inflamed and adherent to the posterior pelvis. All blood cultures and tissue bacterial, mycobacterial, and fungal cultures had no growth. Pathology staining on appendiceal tissue for actinomyces, acid-fast bacilli (AFB) and fungi were all negative. 

**Figure 3:**
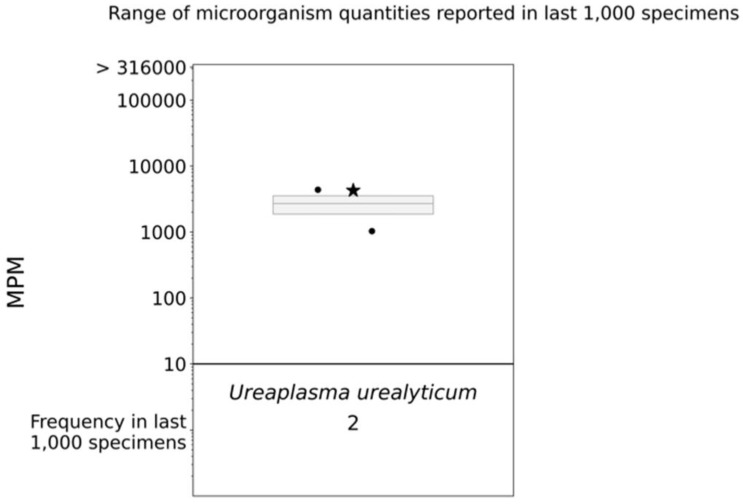
cfDNA mNGS (Karius) boxplot. The star indicates the molecules per milliliter (MPM) of *Ureaplasma urealyticum* identified in our patient’s specimen; the dots indicate the MPM of *Ureaplasma urealyticum* identified in the last 1000 specimens analyzed via Karius.

The patient was started on vancomycin and meropenem. Thereafter, the latter was switched to piperacillin/tazobactam given persistent fever and leukocytosis. Fluconazole and doxycycline were added for empiric atypical bacterial and fungal microorganism coverage. After 12 days of meropenem, but only three days of piperacillin/tazobactam, doxycycline, and fluconazole, the patient defervesced with a resolution of leukocytosis (Figure 1). Urine *Mycoplasma/Ureaplasma* spp. PCR (ARUP Laboratories, Salt Lake City, UT, USA) and tissue broad PCR (The University of Washington, Seattle, DC, USA) were positive only for *U. urealyticum*. Plasma pathogen cell-free DNA metagenomic next-generation sequencing (cfDNA mNGS, “Karius” test) showed 4256 DNA molecules of *U. urealyticum* per microliter (MPM, reference < 10 MPM) with no other organisms detected. In the last 1000 samples tested using Karius, this pathogen was isolated only twice (Figure 3). Concurrent laboratory studies revealed IgG < 320 mg/dL, IgA 33 mg/dL, and IgM 8 mg/dL with zero B-cells on flow cytometry, indicating severe hypogammaglobulinemia (Table 1). She received intravenous immunoglobulin (IVIg) 0.4 g/kg once. In the meantime, she developed acute kidney injury while on vancomycin and piperacillin/tazobactam, which were discontinued and changed to amoxicillin/clavulanate, as she had defervesced after fluconazole and doxycycline were added. She was discharged on the latter three antimicrobials. For her MS, rituximab was discontinued and she was started on twice-daily dimethyl fumarate after discharge. 

Fluconazole was discontinued after two weeks. She completed six weeks of amoxicillin/clavulanate and doxycycline. Amoxicillin/clavulanate was discontinued after tissue PCR returned positive for *U. urealyticum* alone, and she stayed on doxycycline for several months before self-discontinuing. She remains asymptomatic and a repeat CT showed a near-complete resolution of the TOAs. 

In summary, we believe this prolonged and relapsing course was explained by: (1) profound hypogammaglobulinemia from rituximab exposure and (2) partial treatment with short courses of doxycycline, as well as azithromycin and ciprofloxacin early on, with *U. urealyticum* as the dominant pathogen. 

## 3. Discussion

Pelvic inflammatory disease (PID) encompasses a spectrum of disorders involving an ascending infection of the female upper genital tract, including endometritis, salpingitis, and TOAs. With this patient, surgical source control was pursued due to persistent sepsis despite intravenous antibiotics and unsuccessful attempts at less-invasive drainage via interventional radiology. As *U. urealyticum* is susceptible to fluoroquinolones, macrolides, and tetracyclines, the use of empiric doxycycline in the treatment of PID may treat *Mycoplasma* or *Ureaplasma* infection; therefore, their role may be underestimated [14]. In our case, it is possible that empiric initial courses of doxycycline, azithromycin, and ciprofloxacin led to some clinical improvement but were too short to achieve eradication given the patient’s profound hypogammaglobulinemia, and, possibly, need for source control.

*Ureaplasma* species are the smallest self-replicating organisms with a genome ranging between 760 and 1140 kilobase pairs [15]. Despite their small size, *Ureaplasma* species exploit sophisticated virulence factors to establish infection in a host. 

First, *U. urealyticum* produces urease, an enzyme that hydrolyzes urea into carbon dioxide and ammonia, to increase pH in the local environment. This mechanism alters the protective, acidic pH of the vaginal tract maintained by commensal *Lactobacillus* spp. and renders the host more susceptible to infection by other pathogens and/or ascending infection of the reproductive tract [16]. In addition to raising the pH of the local environment, ammonia also irritates mucous membranes to facilitate increased pathogen adherence and colonization. However, the hyperammonemia syndrome seems to have several contributors other than infection [17,18,19], and mainly occurs in patients with impaired renal function, who have elevated blood levels of urea, the substrate of urease for ammonia production [17,19,20]. Our patient had a high-normal ammonia level the only time it was checked, during her second hospitalization. This could be explained by the initiation of doxycycline prior to measuring ammonia level, but is more likely explained by her low blood urea nitrogen level, even when she had acute kidney injury (AKI) (Table 1). 

Second, *Ureaplasma* species establish virulence by producing immunoglobulin A (IgA) proteases. IgA prevents the adherence of microorganisms to the mucosa; thereby, IgA proteases enable invading pathogens to establish infection. This is especially important for ascending infections of the female reproductive tract, as secretory IgA contributes to the protection of vaginal mucosa [21]. 

Third, *Ureaplasma* species express immunogenic surface proteins for cytoadherence. The immunogenic multiple banded antigen (MBA) of *Ureaplasma* species serves to (1) activate complement and pro-inflammatory transcription factors (e.g., NF-kB) to establish an inflammatory disease state and (2) promote attachment to host erythrocytes, neutrophils, urethral epithelial cells, and spermatozoa. MBAs also display antigenic variation, enabling the bacteria to evade the host immune system and persist at sites of invasion [2]. When Mollicute strains are cultured, susceptibility testing should be performed to maximize therapeutic response, given the concern for increasing resistance among *Ureaplasma* spp. [22]. 

The literature on *Ureaplasma* infections signals a strong relationship between humoral immunodeficiency and invasive spread [13,23]. Our case is similar to three prior reports of severe *U. urealyticum* infection in women <30 years old on chronic rituximab therapy for the management of MS (2 cases) [9,13] and granulomatosis with polyangiitis (GPA) (1 case) [10]. Kvalvik et al. (2020) published a case almost identical to ours: a woman in her early twenties with MS, presenting with fever and progressive abdominal pain, was found to have bilateral TOAs [9]. *U. urealyticum* was identified as the causative agent in all case studies through urine culture [13], a PCR of respiratory and urinary samples [10], and pus analysis [9], and was responsive to treatment with doxycycline. Based on our collective findings, we and the authors of the aforementioned studies [9,13] suggest a revision of current guidelines [6] to recommend testing for Mollicute infections in hypogammaglobulinemic patients with PID. 

Iatrogenic (e.g., use of rituximab) or congenital (e.g., common variable immunodeficiency) impairment of humoral immunity predisposes individuals to severe, even disseminated, *U. urealyticum* infection via the weakening of mucosal defenses [23]. This is of particular importance for infections of the genitourinary tract, as IgA plays a key role in its protective immunity. Moreover, *U. urealyticum* exhibits IgA protease activity, further diminishing the humoral immune response to invasion [21]. In a systematic review of disseminated *U. urealyticum* cases between 1989 and 2019, 71% (17/24) of infections occurred in patients with humoral immunodeficiencies [13]. 

Multiplex PCR tests in tissue can be helpful in culture-negative syndromes after the administration of antibiotics, but usually require invasive procedures to obtain tissue samples and the turnaround time (TAT) is quite long (weeks) [24,25,26,27]. In our case, *U. urealyticum* was the only pathogen with DNA identified, which, even after the administration of broad-spectrum antibiotics, strongly supports causality.

The introduction of metagenomic next-generation sequencing (mNGS) of cell-free DNA (cfDNA) (Karius test) is non-invasive, includes a very broad range of pathogens, and has a TAT as short as two business days [28,29,30,31]. This technique is based on sequencing microbial cfDNA via the detection of circulating pathogenic fragments of the genomic DNA throughout the human body, which are found in the purified plasma cfDNA [32,33]. This technology can detect >1000 bacterial, viral, protozoal, and fungal infections, as well as clinically significant polymicrobial infections, often not identified via conventional cultures; therefore, it is frequently referred to as “liquid biopsy” [34,35,36].

To our knowledge, our case is the first whereby the Karius test was used to identify *U. urealyticum* as the most likely culprit pathogen in a case of TOAs with clinically relevant implications for treatment. The diagnostic performance of the test, especially for atypical and rare pathogens, is far from standardized, but three important elements strongly support causality in our case, aside from clinical response to doxycycline. First, the isolation of the same single pathogen via two additional tests, especially multiplex PCR in tissue. Second, the relative high load of plasma cfDNA from an organism that is a known commensal, but is infrequently identified in the blood. Such results with high cfDNA concentrations are unlikely to represent colonization [27]. Third, the identification of a single pathogen in a case of TOA, which is typically considered a polymicrobial infection. Although the Karius test has not been studied in TOAs, the paradigm of febrile neutropenia from mucositis and microbial translocation suggests that cfDNA from multiple pathogens is usually identified in polymicrobial infections, even after the administration of broad-spectrum antibiotics [37,38,39].

## 4. Conclusions

In conclusion, we describe the second case of TOAs caused by *U. urealyticum*, highlighting the pathogenic potential of the Mollicutes in hypogammaglobulinemic patients, and the first of such a case diagnosed by pathogen cfDNA mNGS in plasma. We propose that sexually transmitted infection testing guidelines be revised to include testing for genital mycoplasmas in patients with hypogammaglobulinemia, especially when standard testing is unrevealing. All attempts should be made to collect specimens for analysis directly from the site of infection. However, pathogen cfDNA mNGS analysis of plasma should be considered as a promising non-invasive testing modality with high sensitivity and rapid TAT if an invasive procedure cannot be performed.

## Figures and Tables

**Table 1:** Laboratory values obtained during patient work-up.

Laboratory Test	Value at Admission	Value at Discharge	Reference Range and Units
**Chemistry, Serum**			
Blood urea nitrogen	15 mg/dL	10 mg/dL	6–24 mg/dL
Creatinine	2.57 mg/dL	0.96 mg/dL	0.44–1.03 mg/dL
ALT ^1^	15 IU/L	27 IU/L	6–45 IU/L
AST ^2^	14 IU/L	22 IU/L	10–42 IU/L
Alkaline phosphatase	199 IU/L	152 IU/L	34–104 IU/L
Total bilirubin	0.7 g/dL	0.4 g/dL	0.2–1.3 mg/dL
Albumin	2.4 g/dL	3.3 g/dL	3.5–5.0 g/dL
Total protein	4.8 g/dL	6.5 g/dL	6.0–8.0 g/dL
Ammonia	42 μmol/L	ND ^3^	2–50 μmol/L
**Microbiological Tests**			
*Ureaplasma parvum*, urine PCR	Not detected		Not detected
*Ureaplasma urealyticum*, urine PCR	Detected		Not detected
*Mycoplasma hominis*, urine PCR	Not detected		Not detected
*Mycoplasma genitalium*, urine PCR	Not detected		Not detected
Bacterial detection via PCR with reflex to 16S NGS in blood	*Ureaplasma urealyticum* 4256 DNA MPM		<10 DNA MPM
*Legionella* urine antigen	Negative		Negative
*C. difficile* toxin, PCR	Not detected		Not detected
**Other Tests**			
CRP ^4^	237.33 mg/L	34.78 mg/L	0.00–10.00 mg/L
ESR ^5^	76 mm/h	ND	0–20 mm/h
Urine pregnancy	Negative	ND	Negative
IgG	321 mg/dL	ND	552–1631 mg/dL
IgG_1_	160 mg/dL	ND	240–1118 mg/dL
IgG_2_	99 mg/dL	ND	124–549 mg/dL
IgG_3_	12 mg/dL	ND	21–134 mg/dL
IgG_4_	7 mg/dL	ND	1–123 mg/dL

^1^ ALT (alanine aminotransferase); ^2^ AST (aspartate aminotransferase); ^3^ ND (not done); ^4^ CRP (C-reactive protein); ^5^ ESR (erythrocyte sedimentation rate).

## Data Availability

Data are contained within the article.
